# The relationship between family cohesion and cyberbullying among college students: the chain mediating role of anxiety and cognitive reappraisal

**DOI:** 10.3389/fpsyg.2026.1691755

**Published:** 2026-01-30

**Authors:** Xin Deng

**Affiliations:** Department of General Basic Studies, Criminal Investigation Police University of China, Shenyang, China

**Keywords:** anxiety, cognitive reappraisal, college students, cyberbullying, family cohesion

## Abstract

**Introduction:**

To explore the relationship between family cohesion and cyberbullying behavior among college students, as well as the mediating roles of anxiety and cognitive reappraisal.

**Methods:**

A survey was conducted on 544 college students using the Chinese version of the Family Adaptability and Cohesion Evaluation Scales, the Anxiety Scale, the Emotion Regulation Questionnaire, and the Cyberbullying Scale.

**Results:**

(1) Both family cohesion and cognitive reappraisal showed signiffcant negative correlations with anxiety and college students’ cyberbullying behavior, while anxiety showed a signiffcant positive correlation with cyberbullying behavior. (2) Family cohesion was associated with cyberbullying behavior via the mediating roles of anxiety and cognitive reappraisal, respectively.

**Discussion:**

Anxiety and cognitive reappraisal represent potential psychological pathways through which family cohesion relates to cyberbullying behavior among college students.

## 1 Introduction

While the internet provides convenience for accessing information and communication, it also increases individuals’ exposure to media violence and other harmful information, making them more susceptible to involvement in online victimization events such as cyberbullying (Luo et al., 2023). Cyberbullying behavior refers to an individual’s repeated and intentional acts to harm others through electronic media (Kowalski et al., 2014). With the popularity of social media and instant messaging tools, adolescents are a vulnerable group for cyberbullying (Bauman et al., 2013). However, as one of the main forces of internet use, cyberbullying is also prevalent among college students. Cyberbullying not only causes victims to exhibit behaviors like truancy and skipping class, leading to declining academic performance (Kowalski et al., 2014), but can also lead to psychological crises such as depression, anxiety, and suicidal ideation among victims (Iranzo et al., 2019). The rapid spread, strong anonymity, and difficulty in monitoring make the harm of cyberbullying far exceed that of traditional bullying. Research indicates that the formation mechanisms of cyberbullying include individual factors such as emotion, personality, and moral identity, as well as environmental factors such as school, family, and peers (Buelga et al., 2017; Hu and Xiong, 2024; van Geel et al., 2014). Given the dual threat of cyberbullying to college students’ mental health and the campus ecology, clarifying its mechanisms and developing targeted intervention programs have become urgent practical issues in higher education.

### 1.1 The relationship between family cohesion and cyberbullying among college students

Ecological Systems Theory (Bronfenbrenner, 2005) posits that psychological development results from the interaction between environmental and individual factors. Among environmental factors, the family is a microsystem that directly influences adolescent psychological development. Compared to other family variables (such as parenting styles, parent-child communication, etc.), family cohesion better measures the overall family atmosphere and is a comprehensive indicator reflecting a positive family climate and close relationships among family members (Liu et al., 2014). Family cohesion refers to the degree of emotional bonding among family members; high-cohesion families typically feature open communication, mutual support, and clear boundaries (Olson, 2011). According to Social Learning Theory (Bandura, 1977), adolescents in high-cohesion families learn cooperation, empathy, and non-aggressive conflict resolution strategies through observation and interaction, thereby reducing the likelihood of engaging in bullying in peer or online contexts. The prediction of family cohesion on adolescent cyberbullying is mainly reflected in two aspects: First, parent-child relationships affect the occurrence of bullying. Studies show that adolescents who engage in more bullying generally have incomplete family relationships, lacking effective parental supervision and support (Fanti et al., 2012), while also exhibiting insecure attachment relationships (Eşkisu, 2014). According to Attachment Theory (Bowlby, 1969), adolescents who fail to establish secure emotional bonds with their parents during childhood are more likely to develop negative expectations of others and exhibit more harmful behaviors online to protect themselves. Second, whether parents exhibit bullying behavior in their interactions with others, and their attitudes and ways of handling bullying, also predict adolescents’ construction of cyberbullying (Fang, 2015). Research indicates that good family functioning can predict cyberbullying perpetration among adolescents by affecting interpersonal adaptation (Wang et al., 2020). Therefore, this study proposes Hypothesis 1: Family cohesion can negatively predict cyberbullying behavior.

### 1.2 The mediating role of anxiety

Family functioning, as an external factor for the individual, may not directly affect external behavior; it may exert its influence through other internal individual factors (Zhao et al., 2018). As a primary microsystem environment directly experienced by children, the family is particularly important for children’s psychological adaptation (e.g., anxiety). Family Systems Theory suggests that low family cohesion makes individuals more prone to anxiety in stressful situations by weakening emotional support and a sense of security (Olson, 2011). Previous studies consistently show that family cohesion has a significant negative predictive effect on adolescent anxiety (Bernstein and Borchardt, 1991,^1996^). Deng and Yang’s (2022) survey of 300 college students found that family cohesion explained 39.8% of the variance in social anxiety; lower family cohesion was associated with higher levels of social anxiety. Liu F. et al. (2023) also confirmed a negative correlation between anxiety and family cohesion in a sample of middle school students. Furthermore, Liu X. et al. (2023), in a study of 3,493 primary school students in southwest China, found that family cohesion not only significantly negatively predicted anxiety but also fully mediated this effect through psychological quality. These findings collectively support the “low cohesion → high anxiety” pathway, and this effect shows stability across age groups.

Anxiety is also an emotional experience closely related to aggressive behavior. Highly anxious individuals often exhibit hostile attribution bias and impulse control deficits, thereby increasing the risk of engaging in cyberbullying (Martínez-Monteagudo et al., 2020). It is important to note that the anxiety measure in this study (SAS) assesses state anxiety, reflecting subjective feelings of anxiety over the recent period. Research has shown that such state anxiety is concurrently and prospectively linked to cyberbullying. For example, Giumetti et al. (2022), in a two-wave longitudinal study, found that social anxiety significantly predicted an increase in cyberbullying behavior 6 months later, and this effect remained significant after controlling for depression. Additionally, research on female nursing students also indicated that state-trait anxiety scores were positively correlated with the frequency of cyberbullying perpetration (Alghamdi et al., 2023; Albikawi, 2023), suggesting that gender and professional background did not weaken this association. Overall, anxiety not only coexists concurrently with cyberbullying behavior but can also prospectively exacerbate its occurrence, providing new time-series evidence for the mediating effect of anxiety in the relationship between family cohesion and cyberbullying. Based on this, Hypothesis 2 is proposed: Anxiety mediates the relationship between family cohesion and cyberbullying behavior among college students; lower family cohesion predicts higher anxiety levels, which in turn predicts more cyberbullying behavior.

### 1.3 The mediating role of cognitive reappraisal

Cognitive reappraisal refers to an antecedent-focused emotion regulation strategy aimed at reducing negative emotional experiences by changing the cognitive interpretation of a situation (Gross and John, 2003). Research shows that family cohesion can positively predict the frequency of cognitive reappraisal use among adolescents and secondary vocational students (Song, 2023; Zhao, 2021). Geng et al. (2021), in a sample of 2,801 left-behind children, found that family cohesion was significantly positively correlated with cognitive reappraisal. Furthermore, a survey found that higher family cohesion and adaptability among middle school students were associated with a greater tendency to use cognitive reappraisal as an emotion regulation strategy (You, 2023). In summary, family cohesion not only shows a stable positive correlation with cognitive reappraisal, but this association has been validated in samples across different age groups. Among various emotion regulation strategies, this study focuses specifically on cognitive reappraisal for several key reasons. First, cognitive reappraisal is a well-established, antecedent-focused strategy widely regarded as adaptive and is strongly linked to interpersonal competence and psychological well-being (Gross and John, 2003). Second, there is a solid theoretical and empirical basis connecting the family environment to the development of this cognitive strategy; cohesive families often model and coach cognitive reframing during interactions, thereby fostering its use in children (e.g., You, 2023). Third, cognitive reappraisal has been directly implicated in reducing aggressive behaviors, including cyberbullying (e.g., Jiang et al., 2022; Baroncelli and Ciucci, 2014). While other strategies (e.g., expressive suppression) are important, the present study aimed for a focused examination of this key adaptive pathway.

Research has found that higher levels of cognitive reappraisal are associated with a lower likelihood of college students engaging in cyberbullying (Jiang et al., 2022). Yu (2022) found that cultivating positive emotion regulation strategies can effectively prevent cyberbullying behavior among primary school students. Limited research on cyberbullying also suggests that cyberbullies are less effective at emotion regulation than traditional bullies, as their tendency toward aggressive behavior in cyberspace stems from extreme lack of confidence in their ability to control their emotions (Baroncelli and Ciucci, 2014). He (2025) found a significant negative correlation between emotion regulation and cyberbullying in a sample of Chinese middle school students, indicating that individuals unable to effectively manage uncomfortable emotions may resort to aggressive behavior to try to repair, terminate, or otherwise avoid internal distress. In contrast, individuals with good emotion regulation skills can recognize negative emotions and find acceptable solutions to alleviate or channel them. Therefore, an individual prone to aggressive behavior with low emotion regulation ability is more likely to act aggressively when physiological arousal increases (e.g., during interpersonal conflicts) (Kokkinos and Voulgaridou, 2017). In summary, cognitive reappraisal is not only a protective factor against cyberbullying but may also play a key psychological mechanism in the relationship between family cohesion and cyberbullying. Accordingly, Hypothesis 3 is proposed: Cognitive reappraisal mediates the relationship between family cohesion and cyberbullying behavior among college students; higher family cohesion predicts more frequent use of cognitive reappraisal, which in turn predicts less cyberbullying behavior.

### 1.4 The chain mediating role of anxiety and cognitive reappraisal

The proposed chain mediation posits a sequence from anxiety to cognitive reappraisal. To justify this directional pathway, we draw upon the Emotional Cascade Model (Selby et al., 2008) and contemporary research on the anxiety-emotion regulation interplay. The literature suggests a complex, often bidirectional relationship between anxiety and emotion regulation strategies like cognitive reappraisal, with evidence supporting both regulation strategies as predictors of anxiety symptoms and anxiety as a disruptor of effective regulation (Cisler et al., 2010; Ryan and Eric, 2005). A meta-analysis highlights the importance of distinguishing between trait and state anxiety in these dynamics (Yao et al., 2023).

The Emotional Cascade Model provides a process-oriented framework for our hypothesis. It posits that emotional distress (e.g., elevated state anxiety) can initiate a vicious cycle: negative affect fuels ruminative processes, which intensifies the affective experience, consumes cognitive resources, and impairs the deployment of adaptive regulation strategies, thereby increasing the risk of dysregulated behaviors like aggression.

Applied to our context, we propose that lower family cohesion may increase vulnerability to heightened state anxiety in the face of online interpersonal stressors. This elevated state anxiety can act as the entry point for such a cascade. When confronted with a provocative online interaction, state anxiety may narrow attentional focus onto threat cues and promote ruminative thoughts (e.g., hostile attributions). This cognitively taxing state of anxious rumination is theorized to interfere with the individual’s concurrent capacity to flexibly access and implement cognitively demanding strategies like cognitive reappraisal in that specific moment. Thus, the pathway from state anxiety to cognitive reappraisal in our model reflects a state-dependent regulatory disruption within an episodic cascade, rather than a long-term prediction of trait strategy use. The impaired use of cognitive reappraisal, in turn, leaves negative affect poorly regulated, potentially lowering the threshold for impulsive online aggression such as cyberbullying. Based on this rationale, Hypothesis 4 is proposed: Anxiety and cognitive reappraisal play a chain mediating role in the relationship between family cohesion and cyberbullying behavior among college students; specifically, lower family cohesion is associated with higher state anxiety, which is linked to a reduced likelihood of effectively employing cognitive reappraisal in conflict situations, ultimately relating to more cyberbullying behavior.

## 2 Method

### 2.1 Participants

This survey selected college students enrolled in two universities in Liaoning Province, China. A stratified cluster sampling method was used, with classes as units. Questionnaires were administered during self-study time and collected immediately. A total of 600 questionnaires were distributed, 578 were returned, and 34 invalid questionnaires were excluded, resulting in a final valid sample of 544 participants. Among them, 398 were male (73.2%) and 146 were female (26.8%). There were 147 freshmen (27.02%), 182 sophomores (33.45%), 121 juniors (22.24%), and 94 seniors (12.28%). Three hundred thirty-three participants had urban household registration (61.2%), and 211 had rural household registration (38.8%). Participants’ ages ranged from 18 to 24 years old, with a mean age of 19.70 years (SD = 1.07).

### 2.2 Measures

#### 2.2.1 Chinese version of the family adaptability and cohesion evaluation scales (FACES II-CV)

The second edition of the Family Adaptability and Cohesion Evaluation Scales (FACES II) was developed by Olson et al. (1982). This study used the Chinese version (FACES II-CV) translated by Shen et al. and modified by Wang et al. (1999). This self-report scale consists of two subscales with a total of 30 items. FACES II mainly evaluates two aspects of family functioning: (1) Cohesion, the emotional connection among family members; and (2) Adaptability, the ability of the family system to change in response to problems arising from family situations and different developmental stages. Items were rated on a 5-point Likert scale, ranging from “Never” (1) to “Always” (5). Participants’ responses indicated the extent to which the described situation occurred in their family. In this study, the internal consistency reliability coefficient (Cronbach’s *α*) for the family cohesion subscale was 0.848.

#### 2.2.2 Anxiety scale (SAS)

The Self-Rating Anxiety Scale (SAS) was developed by Zung (1971). Tao and Gao (1994) revised the Chinese version of the Anxiety Scale. This scale is a self-report measure containing 20 items used to assess the subjective feelings of anxiety. The SAS uses a 4-point rating scale, primarily assessing the frequency of defined symptoms: “1” indicates none or a little of the time; “2” some of the time; “3” a good part of the time; “4” most or all of the time. Higher scores indicate higher levels of anxiety. In this study, the internal consistency reliability coefficient for the scale was 0.855.

#### 2.2.3 Emotion regulation questionnaire

The Emotion Regulation Questionnaire developed by Gross and John (2003) and revised by Dong et al. (2008) was used. The Chinese version contains 10 items. All items were rated on a 5-point Likert scale (1 = “Strongly Disagree,” 5 = “Strongly Agree”). Higher scores indicate more frequent use of the corresponding emotion regulation strategy. In this study, the internal consistency reliability coefficient for the cognitive reappraisal subscale was 0.745.

#### 2.2.4 Cyberbullying questionnaire

The Cyberbullying Scale was developed by Wright (2014) and revised by Wang et al. (2016). The Chinese version of the Cyberbullying Scale contains 9 items, rated on a 5-point Likert scale from “Never” (1) to “Always” (5), all scored positively. Higher scores indicate more frequent cyberbullying behavior. In this study, the internal consistency reliability coefficient for the cyberbullying scale was 0.957.

### 2.3 Data analysis

SPSS 26.0 was used for common method bias testing, descriptive statistics, correlation analysis, and exploratory factor analysis (EFA) for item parceling. Mplus 9.0 was used for structural equation modeling (SEM). Given that all constructs were measured using well-validated scales with good reliability in the current sample (see Measures section), the hypothesized structural model was tested directly. To improve model parsimony and the stability of estimates, item parcels were used as indicators for the latent variables (family cohesion, anxiety, cognitive reappraisal, and cyberbullying). Parcels were created separately for each scale based on EFA results, following an item-to-construct balance approach (Little et al., 2002) to distribute item factor loadings evenly across parcels.

## 3 Results

### 3.1 Common method bias test

To avoid common method biases (CMB) in self-report questionnaire analysis, all questionnaires were filled out anonymously to enhance the authenticity of participants’ responses. Harman’s single-factor test was performed using EFA on the 65 items from the four scales. Eleven factors with eigenvalues greater than 1 were extracted, and the first factor explained 21.547% of the variance, which is below the 40% criterion. Therefore, no significant CMB was detected in this study.

### 3.2 Correlation analysis among variables

The results showed significant correlations between all variables except between family adaptability and cognitive reappraisal, which was non-significant (see Table 1).

**Table 1:** Descriptive statistics and correlation matrix for each variable (*N* = 544).

Variable	*M*	SD	1	2	3	4
1. Cohesion	4.44	0.63	1			
2. Anxiety	1.70	0.40	−0.322^**^	1		
3. Cognitive reappraisal	3.42	0.64	0.159^**^	−0.277^**^	1	
4. Cyberbullying behavior	1.34	0.65	−0.167^**^	0.415^**^	−0.256^**^	1

^**^p < 0.01.

### 3.3 Mediating effect analysis

Since family cohesion, anxiety, cognitive reappraisal, and cyberbullying were all latent variables, SEM was employed. The bias-corrected nonparametric percentile Bootstrap estimation method was used for testing, and all variables were standardized. First, the total effect model of family cohesion on cyberbullying was established to test the total effect c and its significance. The results showed that the total effect of family cohesion on cyberbullying was 0.156, and the total effect coefficient was significant (*p* < 0.001). All fit indices were generally good (see Table 2).

**Table 2:** Total effect model and mediating model fit indices.

Model	*χ* ^2^	df	CFI	TLI	SRMR	RMSEA
Total effect model	7.726	4	0.998	0.996	0.010	0.041
Mediating model	70.658	38	0.991	0.987	0.031	0.040

**Figure 1:**
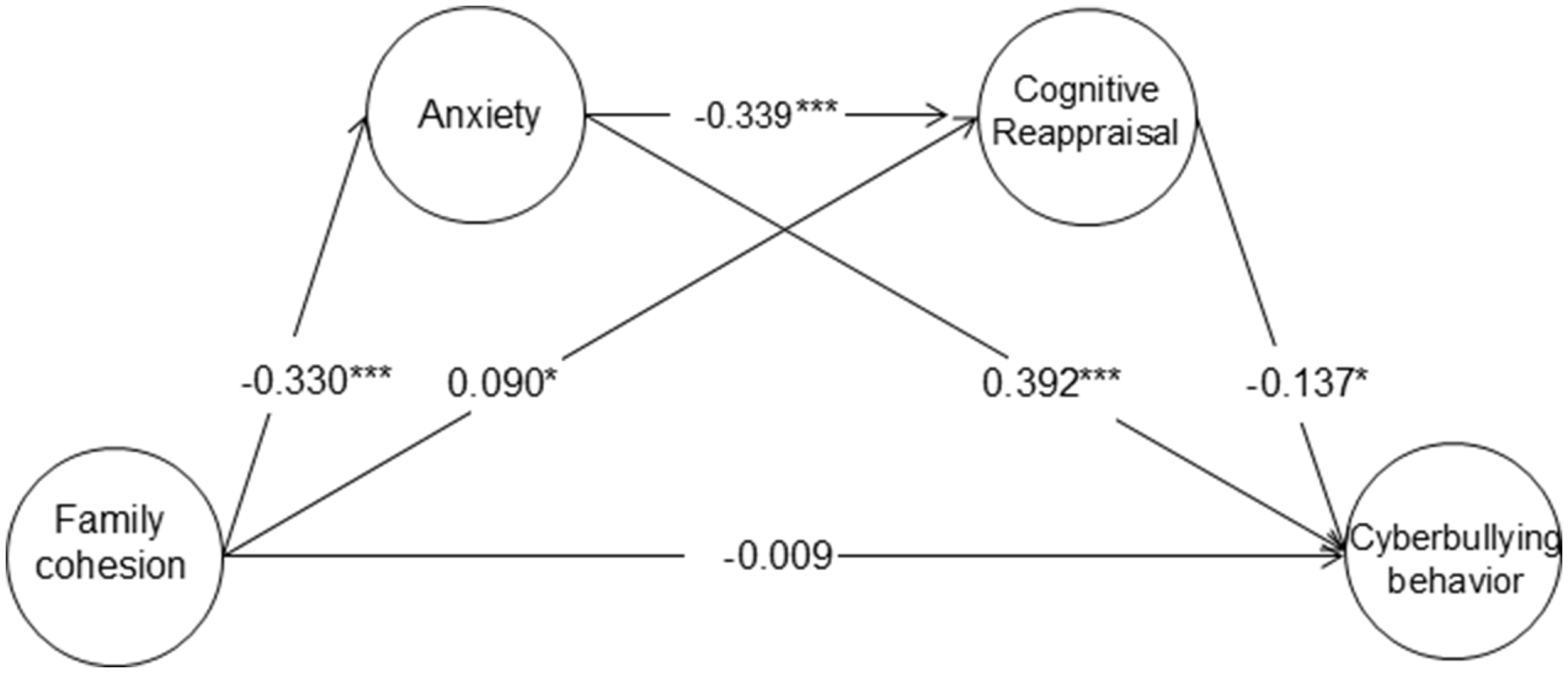
Mediating model of the relationship between family cohesion and cyberbullying behavior. ^*^p < 0.05, ^***^p < 0.001.

Second, the significance of the path coefficients was tested sequentially. In this study, with family cohesion as the independent variable, cyberbullying as the dependent variable, and anxiety and cognitive reappraisal as mediating variables, a mediation model was established (see Figure 1). SEM analysis showed that all fit indices were good (see Table 2), indicating that the model met the standards. Therefore, anxiety and cognitive reappraisal played mediating roles in the relationship between family cohesion and cyberbullying. This mediating effect included three paths: the sole mediating effect of anxiety, the sole mediating effect of cognitive reappraisal, and the chain mediating effect of anxiety → cognitive reappraisal.

Finally, the 95% confidence intervals (CIs) of the path coefficients were estimated with 1,000 bootstrap resamples. The results showed that the mediating effects included two indirect effects: First, the standardized indirect effect 1 generated by the path family cohesion → anxiety → cyberbullying had a 95% CI of (−0.188, −0.064), excluding zero. The mediating effect was 0.129, accounting for 82.69% of the total effect. Second, the standardized indirect effect 2 generated by the path family cohesion → cognitive reappraisal → cyberbullying had a 95% CI of (−0.038, −0.001), excluding zero. The mediating effect was 0.012, accounting for 7.69% of the total effect. Third, the standardized indirect effect 3 generated by the path family cohesion → anxiety → cognitive reappraisal → cyberbullying had a 95% CI of (0.001, 0.016), excluding zero. The mediating effect was 0.015, accounting for 9.82% of the total effect.

When interpreting these indirect effects, it is important to consider both their statistical significance and their magnitude. The bootstrap CIs and the point estimates (e.g., −0.129 for the anxiety pathway) provide information about the effect sizes within our model. The anxiety pathway demonstrated the largest point estimate among the indirect effects. While all indirect paths were statistically significant, their magnitudes vary, which may inform theoretical understanding and potential intervention focus. The proportions of the total effect are sample-specific descriptors and should be interpreted alongside the unstandardized/standardized effect estimates and their clinical or practical context, rather than as standalone indicators of importance.

## 4 Discussion

### 4.1 The relationship between family cohesion and cyberbullying behavior

This study found a significant negative correlation between family cohesion and cyberbullying behavior among college students, consistent with previous research findings (Najjari et al., 2023; Zhao et al., 2018), supporting Hypothesis 1. Social Learning Theory suggests that high-cohesion families provide adolescents with observational learning opportunities for non-aggressive interpersonal scripts through open communication, empathy modeling, and clear conflict resolution rules, thereby reducing the likelihood of them replicating or transferring aggressive behaviors to cyberspace (Bandura, 1977). Furthermore, Family Systems Theory emphasizes that high cohesion enhances individuals’ emotional security and satisfaction of belongingness needs. This “internal working model,” when carried into peer and online environments, can inhibit the formation of hostile attribution bias and revenge motivation (Olson, 2011; Buelga et al., 2017). Therefore, family cohesion is associated with a lower tendency for college students to engage in bullying in online contexts, potentially through dual pathways suggested by social learning and emotional security theories. Future research could introduce more refined family process variables, such as parent-child communication quality and parental internet monitoring strategies, to clarify the specific micro-mechanisms through which cohesion predicts cyberbullying.

### 4.2 The mediating role of anxiety

The results of this study showed that the total effect of family cohesion on college students’ cyberbullying behavior was significant, with 82.69% of the effect size realized through the single mediating path “family cohesion → anxiety → cyberbullying.” This indicates that anxiety plays a mediating role between them, supporting Hypothesis 2. The indirect effect via anxiety (point estimate = −0.129) was not only statistically reliable but also represented the strongest mediating pathway in the model in terms of effect size. This suggests that, within the context of our study, the link between family cohesion and cyberbullying is substantially explained by anxiety. From a practical standpoint, this underscores anxiety as a critical potential target for intervention. Even modest reductions in anxiety, theoretically facilitated by enhancing family cohesion, could be associated with meaningful decreases in the risk of cyberbullying perpetration, given the strength of this mediating pathway.

Deng and Yang (2022) found that family cohesion and adaptability among college students explained 39.8% of the variance in social anxiety; lower levels of family cohesion and adaptability were associated with higher levels of social anxiety. Family cohesion reflects the emotional connection among family members. Families with low cohesion are prone to conflicts and disharmony, easily leading children to lack needs for love, respect, and security. Problems inevitably exist in communication and daily interaction patterns among family members. According to Bandura’s Observational Learning Theory, within the family, all members are models for children to imitate and learn from. In such an environment, children internalize the family’s interpersonal communication patterns as their own patterns for interacting with others. These maladaptive patterns can cause problems and trigger anxiety when interacting with others. Anxiety, in turn, significantly positively predicts cyberbullying behavior among college students (Jia et al., 2021). This finding is consistent with a theoretical pathway where lower family cohesion is linked to higher state anxiety, which in turn is associated with higher levels of cyberbullying. This finding aligns well with the perspectives of Emotional Security Theory (Davies et al., 2006) and the Emotional Cascade Model (Selby et al., 2009): low family cohesion weakens children’s sense of security within the family subsystem, thereby activating anxiety responses characterized by hypervigilance and hostile attribution. Highly anxious individuals, due to depleted impulse control and emotion regulation resources, are more likely to engage in aggressive behavior in online contexts.

### 4.3 The mediating role of cognitive reappraisal

The SEM showed that 7.69% of the effect of family cohesion on cyberbullying was realized through the sole mediating path “family cohesion → cognitive reappraisal → cyberbullying.” The 95% bootstrap CI did not include zero, indicating that the mediating effect of cognitive reappraisal was significant, supporting Hypothesis 3. Previous research found that children raised in families with high cohesion and high adaptability tend to use cognitive reappraisal for emotion regulation, whereas children from families with low cohesion and low adaptability may rely more on expressive suppression (You, 2023). High-cohesion and adaptable family environments, by providing emotional support and open communication patterns, model for individuals how to cope with social conflicts through cognitive restructuring. For example, family members tend to use strategies like “perspective-taking” or “positive attribution” when facing conflicts. This interaction pattern is internalized by children through observational learning and then transferred to peer relationships (Bandura, 1977). In this study, the predictive effect of family cohesion on cognitive reappraisal further indicates that the family is not only a place for emotional bonding but also a crucial environment for practicing emotion management skills.

The core reason why an individual’s cognitive reappraisal strategy can effectively reduce cyberbullying behavior lies in its ability to adjust the individual’s cognitive interpretation of emotional events in online interactions, thereby weakening the psychological motives for bullying (such as anxiety) at their root and enhancing self-regulation ability. When individuals receive potentially conflict-inducing information (e.g., criticism or teasing from others), cognitive reappraisal guides them to actively consider alternative explanations like “the other person might have made an unintentional mistake” rather than directly attributing it to “hostile attack.” Therefore, cognitive reappraisal can enhance an individual’s empathy and problem-solving abilities by reducing defensive reactions in interpersonal interactions (Ke and Yang, 2025), thereby significantly reducing cyberbullying (Tang and Liu, 2024).

The discovery of this pathway provides empirical support for the integration of Family Systems Theory and Emotion Regulation Theory. It suggests that high-cohesion families, by providing a secure emotional atmosphere and positive emotional coaching, promote children’s priority use of adaptive regulation strategies (like cognitive reappraisal) when facing peer or online pressure, thereby reducing hostile and impulsive reactions. Overall, as an antecedent-focused emotion regulation strategy, cognitive reappraisal plays the role of a “psychological buffer” between family cohesion and cyberbullying. Therefore, enhancing college students’ reappraisal ability may be a feasible intervention target for preventing cyberbullying at the family system level.

### 4.4 The chain mediating role of anxiety and cognitive reappraisal

This study further found that family cohesion could predict college students’ cyberbullying behavior through the chain path “anxiety → cognitive reappraisal.” This chain mediating effect accounted for 9.82% of the total effect, and the Bootstrap 95% CI (0.001, 0.016) did not include zero, indicating a significant chain effect and supporting Hypothesis 4. This finding can be best interpreted through the lens of the Emotional Cascade Model (Selby et al., 2008), which provides a process-oriented framework for understanding how discrete emotional experiences can escalate into dysregulated behavior. Consistent with our theoretical framing in the introduction, we posit that individuals from less cohesive families may be more susceptible to experiencing heightened state anxiety when confronted with online interpersonal stressors (e.g., perceived provocation or conflict). This elevated state anxiety can initiate a cascade by fueling ruminative processes (e.g., hostile brooding about the slight), which narrows cognitive focus and consumes critical mental resources. It is this state of anxious rumination that interferes with the concurrent, effortful cognitive operations required to flexibly generate and implement alternative appraisals of the situation—that is, to engage in cognitive reappraisal effectively in that specific moment. The failure to deploy this adaptive regulatory strategy leaves the intensified negative affect unmodulated, thereby lowering the threshold for impulsive, aggressive online actions such as cyberbullying, which may serve as a maladaptive attempt to distract from or terminate the aversive emotional cascade. Therefore, the identified chain pathway likely captures a snapshot of this proposed episodic sequence, wherein state anxiety triggers a ruminative cycle that momentarily disrupts the situational efficacy of cognitive reappraisal, facilitating dysregulated behavioral discharge. From an intervention perspective, this model highlights the potential utility of integrated strategies that not only aim to reduce anxiety but also train individuals to disrupt the anxiety-rumination link and bolster cognitive reappraisal skills for use under stress.

### 4.5 Research limitations and future directions

This study is the first to test the chain mediation model “family cohesion → anxiety → cognitive reappraisal → cyberbullying” in a sample of Chinese college students, providing new empirical evidence for understanding the family-emotion mechanisms of cyberbullying. However, the following limitations remain and need to be addressed and expanded upon in future research. First, the cross-sectional design cannot establish causal relationships between variables. Future research could adopt multi-wave longitudinal sampling to establish stricter causal sequences among family cohesion, anxiety, cognitive reappraisal, and cyberbullying, verifying the persistence of the chain effect and potential bidirectional influences. Second, all data in this study came from self-report questionnaires, which may be affected by social desirability bias and CMB. Harman’s single-factor test is a widely used diagnostic technique; however, it has recognized limitations, including sensitivity to the number of factors extracted and the assumption that a single factor can account for most method variance. More robust statistical controls for CMB, such as the latent method factor approach or marker variable technique, were not employed in this study but represent valuable avenues for methodological rigor in future research. Third, the sample was mainly from two universities, with a notable gender imbalance (73.2% male). Given established gender differences in cyberbullying (e.g., prevalence, forms) and emotion regulation strategies, this imbalance may limit the generalizability of the findings, particularly to female college students. In this study, gender was not included as a control variable or moderator in the primary analysis, as our aim was to test the theoretical model in the overall sample. Future research should explicitly test for gender differences, consider gender as a potential moderator, and strive for more balanced and diverse samples to enhance external validity and understand potential gender-specific mechanisms.

## 5 Conclusion

Using a sample of 544 Chinese college students, this study systematically examined the relationship between family cohesion and cyberbullying behavior and its underlying psychological mechanisms. The results indicated that: (1) Family cohesion was significantly negatively correlated with cyberbullying. (2) Anxiety and cognitive reappraisal each separately mediated this relationship, with anxiety having the largest mediating effect size. (3) The chain path “anxiety → cognitive reappraisal” constituted a significant chain mediating effect, revealing a potential cascade process consistent with theory: lower family cohesion was linked to higher anxiety, which was associated with lower cognitive reappraisal, ultimately relating to higher cyberbullying behavior. The study confirms the integrative value of family systems, emotional dysregulation, and emotion regulation strategies in explaining cyberbullying among college students, providing a theoretical basis for universities to develop multi-component intervention programs centered on “enhancing family communication—alleviating anxiety—training cognitive reappraisal.”

## Data Availability

The raw data supporting the conclusions of this article will be made available by the authors, without undue reservation.
